# Clinical pharmacists in primary care general practices: evaluation of current workforce and their distribution

**DOI:** 10.1186/s40545-022-00483-3

**Published:** 2022-12-09

**Authors:** Elisha Chopra, Tanvi Choudhary, Ankie Hazen, Sunil Shrestha, Inderpal Dehele, Vibhu Paudyal

**Affiliations:** 1 School of Pharmacy, College of Medical and Dental Sciences, University of Birmingham, Birmingham, UK; 2 Julius Center for Health Sciences and Primary Care, University Medical Center Utrecht, Utrecht, The Netherlands; 3 School of Pharmacy, Monash University Malaysia, Jalan Lagoon Selatan, 47500 Bandar Sunway, Selangor Malaysia

**Keywords:** Clinical pharmacist, Primary care, General practice, Physician offices, Workforce

## Abstract

**Background:**

General practices in primary care across England are increasingly employing clinical pharmacists to help tackle the workforce crisis and alleviate pressure. Clinical pharmacists can provide administrative and clinical duties, including non-medical prescribing, advice on polypharmacy and medicines optimisation. The aim of this study was to investigate the distribution of clinical pharmacists in general practice across England, and explore the relationship between the distribution and regional demography.

**Methods:**

This study used publicly available government database from various sources pertaining to primary care general practice workforce and population demographics of England. The number and distribution of pharmacists working within general practices in England were analysed and compared across practices considering general practitioner (GP), nurse and patient population in the practices, patients age ≥ 65 years and over and the Index of Multiple Deprivation (IMD) scores.

**Results:**

Twenty two percentage (1469 of 6674) of practices in England were found to have access to a clinical pharmacist, equating to 1358 full-time equivalent (FTE) pharmacists and a mean pharmacist FTE of 10.07 (95% CI 8.40, 11.75, SD = 9.84) per Clinical Commissioning Group (CCG). A significant relationship between pharmacist FTE and the number of patients 65 years and older [*r* (132) = 0.75, *P* < 0.001)] was observed; however, the distribution was not related to population deprivation scores.

**Conclusions:**

Approximately one in five general practices in England have access to a clinical pharmacist. Further research is needed to ensure wider and equitable distribution based on workforce needs and practice population demography.

**Supplementary Information:**

The online version contains supplementary material available at 10.1186/s40545-022-00483-3.

## Background

It has been suggested that the general practice workforce in England has been facing a crisis. Currently, more than 50% of general practitioners (GPs) are over the age of 50 [1]. An aging population coupled with multi-morbidity contributes to the unprecedented pressure that primary care is facing [2]. To alleviate pressures that GPs face and provide a multi-skilled task force, clinical pharmacists are increasingly being employed within general practices.

The ‘Clinical Pharmacists in General Practice’ scheme was introduced as part of the Five Year Forward Review by the National Health Service (NHS) England to address the staff shortage in general practice in 2015 [3]. The £15 million scheme aimed to support the expansion of the general practice workforce, which included the employment of clinical pharmacists [4]. NHS England was responsible for approving applications through this scheme which saw the employment of 460 clinical pharmacists in the first phase of the scheme [5]. NHS England later expanded the scheme with an additional funding of £112 million for a further 1500 clinical pharmacist posts that was later terminated in April 2019 [6]. In July 2019, a new Network-Contracted Directed Enhanced Service (DES) was initiated as part of the new GP 5-year contract framework to support Primary Care Networks (PCNs) with the recruitment of an additional 20,000 staff to work in primary care, including clinical pharmacists [6]. This initiative is set to take over the ‘Clinical Pharmacist in General Practice’ scheme.

Pharmacists have a range of responsibilities within practices, from providing clinical services to performing administrative duties. Clinical services that pharmacists provide include operating minor ailment clinics, face-to-face polypharmacy medication reviews, managing and prescribing for long-term conditions and addressing medication adherence [7]. The role of a pharmacist also includes: providing education, dealing with hospital discharge letters, detecting and managing adverse drug reactions (ADRs), overseeing audits and being a point of contract for medicine-related enquiries [7]. By taking on routine tasks, pharmacists are increasing the capacity of general practices, thereby increasing patient access, reducing prescribing errors and increasing strategic prescribing [8].

The integration of clinical pharmacists into general practices has proven beneficial in improving patient care and multidisciplinary work. A systemic review conducted by Tan et al. [9] found that 86.8% of pharmacist interventions included medication reviews. The study included 19 studies, which found that the pharmacists’ intervention had positive clinical outcomes, and primary outcomes related to medication use. The finding of the meta-analysis favoured pharmacist interventions with significant improvement in physiological parameters observed compared to control patients [9].

In 2015, a pilot scheme was commissioned by NHS England to explore the impact of clinical pharmacists, describe how they affect working practices and improve service delivery [10]. From the pilot, 98% of pharmacists undertook patient-facing roles, focusing on complex medication reviews, polypharmacy and deprescribing [10]. In addition, 38% of pharmacists reported undertaking medication reviews daily as part of their role [10]. The pilot scheme concluded that clinical pharmacists contribute to an increase in general practice capacity, which was cited as the main benefit of the scheme.

Despite increasing clinical pharmacists working as part of the general practice team, the extent of their service provision and their distribution in general practice is still unknown. The aim of this study was to investigate the distribution of clinical pharmacists in general practice across England and explore the relationship between the distribution and regional demography.

## Methods

### Study design

This study undertook a secondary analysis of quarterly collected general practice data, concentrating on the clinical pharmacist workforce in general practices within NHS England primary care settings and geographical areas with varying socioeconomic status.

### Ethics approval

Ethical approval was not obligatory as data extracted for this study were retrieved from publicly available UK government data sets. None of the general practices, pharmacists or patients were identified in the datasets or this manuscript.

### Data collection and analysis

Analysis of routinely collected government data from online databases was undertaken. NHS digital sources were consulted, and the ‘General Practice Workforce’ data set was extracted, which presented workforce data at practice level [11]. From this data set, the headcounts and Full Time Equivalent (FTE) were retrieved for pharmacists, doctors and nurses. One FTE equates to 37.5 contracted working hours [12]. A total number of patients and those aged 65 years and over were also collated due to this patient demographic often presenting with multiple co-morbidities and polypharmacy [12]. Practices that did not provide pharmacist numbers were excluded from the analysis. The pharmacist workforce was analysed at practice, clinical commissioning group (CCG), and regional level to understand pharmacist numbers and their distributions.

Incidence of Multiple Deprivation (IMD) for each of the Clinical Commissioning Groups (CCGs) was extracted [13]. CCGs are statutory NHS bodies responsible for planning and authorising health services for their vicinity [14]. The 135 CCGs were ranked in accordance with their corresponding IMD average score and split into deprivation quintiles with equal numbers of CCGs in each group to explore the link between deprivation and clinical pharmacist adjusted for populations [15].

All data were extracted and checked independently for accuracy. IBM Statistical Package for Social Sciences (SPSS) version 26.0 for Windows (IBM Corporation, Armonk, NY, USA), quantitative was used for data analysis. Descriptive analysis using frequencies and percentages was conducted to determine the capacity of the workforce. Bivariate correlation analysis was conducted using continuous variables to determine the association between the dependent variables (pharmacist headcount and FTE with or without population adjustment) and dependent variables (GP and nurse headcount, FTE and patient populations). Pearson’s correlation was the chosen method of correlation conducted, with significance tested at *α* = 0.01. Multiple regression was conducted. The influences of categorical variables on pharmacist supply were tested using one-way ANOVA. This included testing the differences in pharmacist supply between deprivation quintiles and of the Health Education England (HEE) regions. If a statistically significant difference were observed between groups following ANOVA, a post-hoc test would be conducted.

The reporting conforms to The REporting of studies Conducted using Observational Routinely-collected health Data (RECORD) Statement (Additional file 1).

## Results

Overall, 6674 general practices in England provided workforce data. Out of which, 324 were excluded from analysis as pharmacist numbers were not reported. In total, 22% of all general practices who reported the workforce data in England employed a clinical pharmacist as part of their multidisciplinary team. On average, general practices had a mean FTE clinical pharmacist of 0.21 (95% CI 0.20, 0.23, SD = 0.53), corresponding to an average of 8 contracted hours per week, per practice (Table 1). A mean of 14.65 (95% CI 12.34, 16.96, SD = 13.56) clinical pharmacists were employed per CCG, equating to a mean FTE of 10.07 (95% CI 8.40, 11.75, SD = 9.84) per CCG. In total, 1981 clinical pharmacists were employed as part of the NHS reimbursement scheme, equalling a 1358 FTE (Table 1).

**Table 1:** Workforce statistics of clinical pharmacists in general practices in England presented at practice and clinical commissioning group (CCG) regions

	Pharmacist headcount		Pharmacist headcount per 10,000 population		Pharmacist FTE		Pharmacist FTE per 10,000	
	Mean	S.D	Mean	S.D	Mean	S.D	Mean	S.D
General practice level	0.31	0.69	0.31	0.78	0.21	0.53	0.19	0.53
CCG level	14.65	13.56	0.34	0.21	10.07	9.85	0.23	0.15

**Fig. 1:**
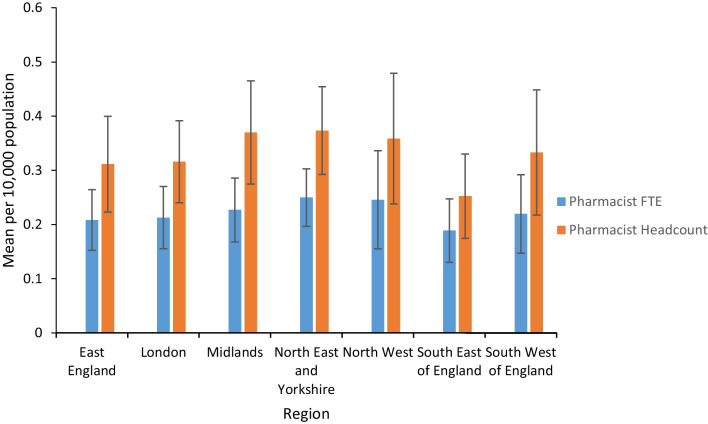
Mean pharmacist headcount and full time equivalent (FTE) adjusted for population within English regions

Figure 1 presents workforce statistics adjusted for each region within England. Average FTE per 10,000 patients ranged from 0.19 (95% CI 0.13–0.25) FTE in the South East of England to 0.25 (95% CI 0.20–0.30) FTE in the North East and Yorkshire. The data set demonstrated no statistically significant difference in pharmacist FTE between regions as determined by one-way ANOVA, *F* (6, 128) = 0.44, *P* > 0.001. Furthermore, pharmacist headcount ranged from a mean of 0.25 (95% CI 0.17–0.33) per 10,000 patients in the South East of England to 0.37 (95% CI 0.29–0.45) in the North East and Yorkshire. Likewise, no statistically significant difference in pharmacist headcount between regions was established by one-way ANOVA, *F* (6, 128) = 0.83, *P* > 0.001.

### Deprivation analysis

**Fig. 2:**
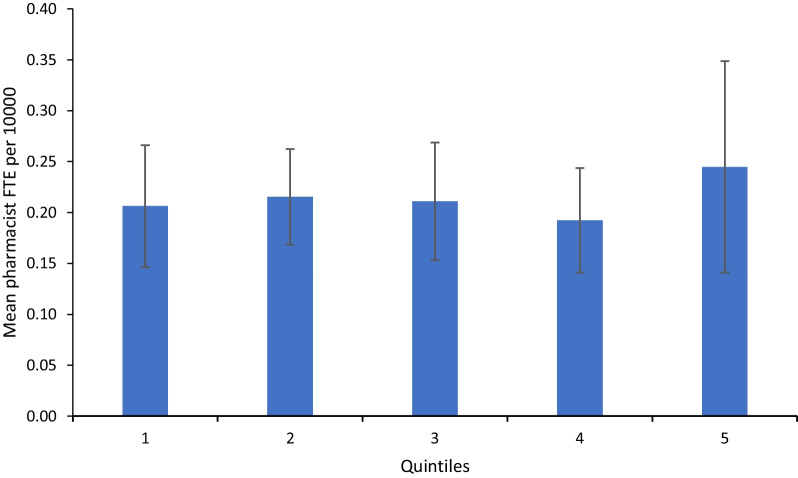
Mean pharmacist full time equivalent (FTE) adjusted for population by deprivation quintiles

Data from 135 CCGs in England were available from the NHS digital database. Of these 135 CCGs, the English Indices of Deprivation (IMD) data for England were available for 108 CCGs in the form of an IMD average score. CCGs were ranked into one of five deprivation quintiles based on their scores. Quintile 5, the most deprived quintile, had a mean pharmacist FTE of 0.24 (95% CI 0.14–0.35) per 10,000 patients. Quintile 1, the least deprived, had a mean pharmacist FTE of 0.21 (95% CI 0.15 to 0.27) per 10,000 patients. No statistically significant difference in pharmacist FTE between quintiles was determined as seen by one-way ANOVA analysis, *F* (4. 102) = 0.36, *P* > 0.01 (Fig. 2).

### Correlation analysis

**Fig. 3:**
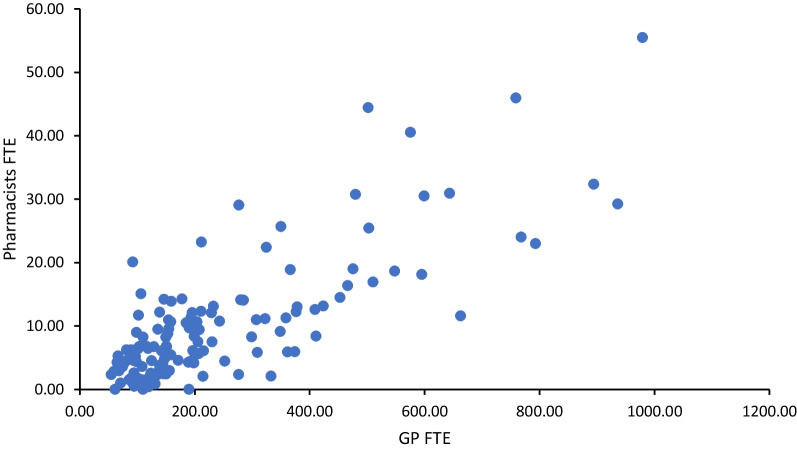
Pharmacist FTE by GP FTE of all English CCGs *r*(132) = 0.79, *P* < 0.001

**Fig. 4:**
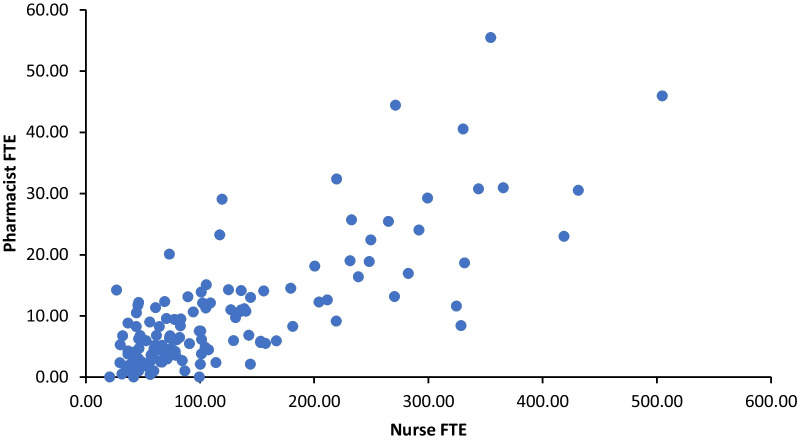
Pharmacist FTE by Nurse FTE of all English CCGs, *r*(132) = 0.78, *P* < 0.001

Figures 3 and 4 describe the association between pharmacists’ FTE and the variables, GP FTE and nurse FTE. Bivariate analysis detailed a significant association between the total pharmacists FTE at CCG level and total GP FTE (Pearson’s *r* = 0.79, *P* < 0.001) and nurse FTE (Pearson’s *r* = 0.78, *P* < 0.001) at the corresponding CCG level. A positive association between pharmacist FTE and GP FTE and nurse FTE was also observed.

**Fig. 5:**
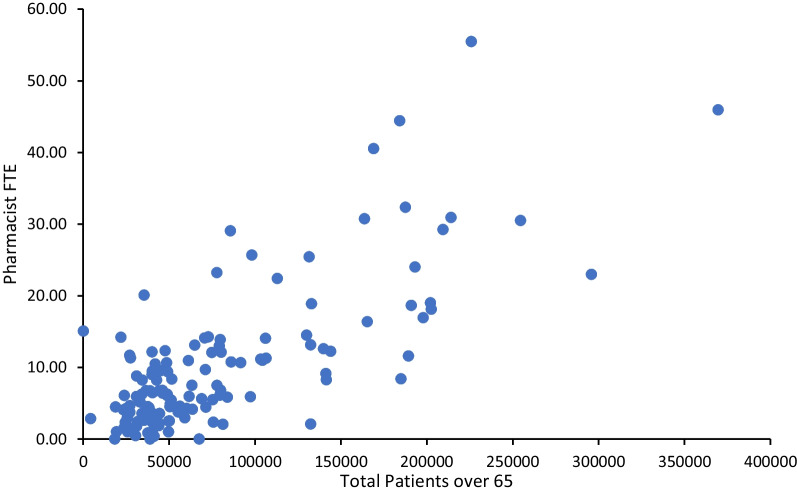
Pharmacist FTE by total patients over the age of 65 of all English CCGs, *r*(132) = 0.75, *P* < 0.001

Figure 5 details a bivariate analysis exhibiting a positive association between pharmacist FTE and the total number of patients over the age of 65, with both variables increasing in respect to each other. Pearson’s product–moment correlation determined the association to be statistically significant (Pearson’s *r* = 0.75, *P* < 0.001).

In addition, multiple regression analysis investigated the significance of GP FTE, nurse FTE and patients over 65 numbers on pharmacist FTE. The culmination of these variables accounted for 66% of the variance in pharmacist FTE. Overall, the regression model and all three variables were statistically significant to the prediction of pharmacist FTE, *F*(3,131) = 85.15, *P* < 0.001, *R*^2^ = 0.66.

## Discussion

### Summary and discussion of key findings

The aim of this study was to investigate the current clinical pharmacist workforce in general practices across England, and to examine characteristics associated with their employment. A total of 22% of general practices in England who reported their workforce data have access to a clinical pharmacist.

The analysis demonstrated no significant relation between the regional variations in pharmacist numbers and FTEs. In addition, no relationship was determined between deprivation and the availability of a pharmacist despite the greater need for primary care services in more deprived areas [16]. Regarding practice characteristics, a positive association was observed between GP and nurse FTE and pharmacist FTE. A positive correlation was also true for pharmacist FTE and the total number of patients over the age of 65. Patients over the age of 65 are more likely to have more complex care needs and multi-morbidity [16]. Therefore, variation in the distribution of pharmacists in general practice may be associated with the workforce composition of general practices and their patient demography. The subsequent workload of practices, CCGs and patient needs may be a strong conjecture of pharmacist distribution rather than the wider demography of the patient population.

### Strengths and limitations

To our knowledge, this is the first study analysing the pharmacy workforce in general practices in England, particularly in the context of population demography. This study used an established workforce data set. General workforce data ARE collected quarterly; therefore, staff in general practice should be familiar with the reporting procedure. The national data set, monitored by NHS digital, provided robust details of the general practice workforce in England as information regarding workforce headcount and FTE was described. General practice workforce statistics were collated using two data sources, including the workforce Minimum Data Set (wMDS) provided directly from general practice via the National Workforce Reporting System (NWRS) data entry module [18]. This provided information on all staff working in practice, excluding GP Registrars. The NWRS further included figures from Health Education England (HEE) regions, making it the main data source for the general practice workforce. However, these data sets offered estimations for headcount and FTE for practices that did not provide complete and/or valid data; this could be due to poor data quality or not submitted data [17]. The data set also provided information on patient demography included in the analysis. The use of multiple data sources and variables allowed for the adoption of multivariable statistical models. In doing so, independent variable effects can be observed on pharmacist headcount and FTE.

However, the completeness of the workforce data may be compromised as a result of the COVID-19 pandemic. Due to increased pressure on the general practice workforce at the time, not all practices may have had the opportunity to update their NWRS data in time for the extraction. Consequently, disruption may have resulted in under-reporting, and as a result, NHS digital stated fewer new records than expected [17]. However, practices that failed to disclose pharmacist numbers were excluded from the analysis.

Furthermore, the completeness of pharmacist headcounts and FTE may be compromised as the data only accounted for pharmacists employed in general practice as part of the Network Contract Directed Enhanced Services (DES), a scheme which only accounts for 70% of the employment costs for clinical pharmacists as of 2019 [18]. Therefore, other ways that pharmacists could be recruited into general practice were omitted. Most notably is the recruitment of clinical pharmacists via Primary Care Networks (PCNs) [18]. PCNs include general practices working alongside other health, social care, metal health and voluntary sector providers to provide integrated services to local communities. Therefore, PCNs have a distinct workforce, solitary to that of the general practices and CCGs that were not included in the General Practice Workforce data set. Pharmacists employed via PCNs may, therefore, mean that pharmacists contracted working hours could be split across multiple practices and CCGs. Hence, the FTE is not included in the database [17]. As the working hours of pharmacists are transferred to local PCNs, the true FTE of a clinical pharmacist in practice may not be equivalent to the one present in the given data set [17].

In addition, whether locum pharmacists were included in the headcount was not specified. The data also did not provide information as to how long employed pharmacists have been qualified, the role undertaken by the pharmacist within the practice and what qualifications the pharmacists employed held (for instance, independent prescribing qualifications). With an independent prescribing qualification, the role that a pharmacist could adopt as part of the multidisciplinary team would be different from that of a pharmacist who could not prescribe. A cross-sectional survey of the pharmacy workforce in general practices in Scotland revealed two-thirds of pharmacists employed within general practices are independent prescribers, with three-quarters of pharmacists undertaking prescribing activities [19]. Hence, a true representation and extent to which pharmacists are integrated, their roles and qualifications in general practices in England are currently unknown.

### Implications for practice and research

This paper demonstrates that the supply and distribution of pharmacists in general practices are determined by workload and patient demography of practices rather than the associated deprivation. With general practice collaborative reforms set to introduce a mixed skill workforce into general practice, recruitment of clinical pharmacists is likely to increase to help alleviate the pressures that general practices and the NHS face [3]. The total number of clinical pharmacists in general practice are now at 2800, and thousands more are due to be appointed by 2024 under the NHS Long-Term Plan. The findings show that pharmacists tend to be in greater numbers in general practices with a higher proportion of older adult populations who are more likely to have complex care needs and be at risk of inappropriate polypharmacy.

The impact of pharmacists in general practice has been extensively documented. Existing literature identifies the benefits of pharmacists in general practices, with GPs valuing the medication expertise pharmacists provide [20]. There is also agreement that pharmacists relieve workload pressures in general practice [21]. A systematic review further revealed a reduction in medication-relation errors and improvements in the clinical outcome of patients, most notably in cardiovascular disease and diabetes [9]. Moreover, observations have seen an additional benefit of cost-effectiveness due to pharmacist interventions [11]. Finally, an inclusion of clinical pharmacists, who can prescribe within general practice, can release an average of 5 h of direct GP time per week, adding further value to the multidisciplinary team [8]. Consequently, pharmacists with prescribing qualifications have a positive impact on alleviating general practice pressure.

Additional exploration of variables such as the number of patients with multi-morbidity and polypharmacy at practice level could be linked with findings to further understand pharmacist workload and if these variables influence the distribution and supply of clinical pharmacists in general practices. Further research is needed to explore the views of GPs, other general practice staff, stakeholders, CCG and PCN members as to how clinical pharmacists are contributing to the workforce. The importance of role description, training pathways, prescribing protocols and funding were emphasised in a study of key stakeholders in relation to strengthening the roles of pharmacists in a recent study [22]. Patients were shown to have expressed high satisfaction with general practice pharmacists’ advice in a survey conducted across general practices in the South–East of England [23].

To achieve equitable distribution of pharmacists in general practices, recruitment should ideally consider both deprivation and patient demography. It is known that pharmacies and hence pharmacist workforce in the community is positively distributed with deprivation, with areas of higher deprivation having proportionately higher number and hence greater access to community pharmacies [24]. Pharmacists in the community provide services that aim to address inequality of care and to alleviate health impact of deprivation, including provision of minor ailment services, substance misuse service and medication reviews [25–27]. Innovative services to offer person-centred and integrated care to the most disadvantaged in the community, such as those with dual diagnosis of substance misuse and mental health problems, learning disabilities and persons experiencing homelessness are essential [28–30]. Similarly, there is opportunity for the general practice based pharmacist workforce to offer services to address wider determinants of health. In particular, offering medication reviews to target groups with multimorbidity, addressing inappropriate polypharmacy, including deprescribing of medicines such as opioids and benzodiazepines, provision of home health and outreach services, patient education and counselling, prescriber education in medicines optimisation are some of the services that are likely to lend to positive outcomes.

With the role of a pharmacist in general practice expecting to expand, the education and training of pharmacists must reflect these changes. The introduction of a Foundation training year in 2021, which is set to replace the pharmacy pre-registration year, aims to prepare pharmacists prescribers by the end of the programme [31]. Educational reforms would further help alleviate pressures that general practice and the NHS as a whole are facing. Pharmacists in the community can also ease pressure on other primary care areas by prescribing, supplying and optimising of patient medications for long term health conditions [32–34] as well as offering services to mitigate health inequalities [35–39].

## Conclusion

Clinical pharmacists are increasingly contributing to the general practice workforce. The introduction of clinical pharmacists into general practice is a rapidly progressing role, introduced to help tackle the shortage of GPs and reduce the consequences of this. This study shows that just over alone in five general practices in England have access to a clinical pharmacist. Such pharmacists are predominantly situated in practices with high proportions of patients aged of 65 and over. Overall, deprivation did not significantly impact the availability of a pharmacist. Further research is needed to identify and strengthen their current roles in supporting the GP workforce and ensuring equitable distribution per population demography.

## Supplementary Information

**Additional file 1.** The RECORD statement—checklist of items, extended from the STROBE statement, that should be reported in observational studies using routinely collected health data.

## Data Availability

All data in relation to this study are presented in this manuscript.

## Abbreviations

ADRs: Adverse drug reactions
CCG: Clinical Commissioning Group
DES: Directed Enhanced Service
FTE: Full-time equivalent
GPs: General practitioners
HEE: Health Education England
IMD: Index of Multiple Deprivation
NHS: National Health Service
NWRS: National Workforce Reporting System
PCNs: Primary Care Networks
SPSS: Statistical Package for Social Sciences
RECORD: The REporting of studies Conducted using Observational Routinely-collected health Data
wMDS: Workforce Minimum Data Set

## Declarations

### Permission to reproduce material from other sources

The data contained in this manuscript have been obtained from public domains hosted by UK Government databases.
